# Supplementary material for: Patterns of Intron Gain and Loss in Fungi
Source: PLoS Biol. 2004 Nov 30;2(12):e422. doi: 10.1371/journal.pbio.0020422 (PMC532390; doi:10.1371/journal.pbio.0020422)
Supplement: Table S1 — Also available at http://genes.mit.edu/NielsenEtAl/. (4.3 MB ZIP). [file pbio.0020422.st001.zip › NielsenEtAl/html/568.html]

AN0410.1.NCU00043.1.MG10195.1.FG07233.1


```
 CLUSTAL W (1.82) Multiple Sequence Alignments - Introns Inserted


Sequence 1: MG10195.1	325 aa
Sequence 2: FG07233.1	303 aa
Sequence 3: NCU00043.1	310 aa
Sequence 4: AN0410.1	323 aa
Alignment Length: 326 aa
Number Identitical Residues: 288 aa
Alignment Score (without introns) 11367


MG10195.1 	MADQHDVDLDSIIDRLLEVRGSRPGKQVQLLEAEIRYLCTKAREIFISQPILLELEAPIK
NCU00043.1	MADHTEVDLDSIIDRLLEVRGSRPGKQVQLLEAEIRYLCTKAREIFISQPILLELEAPIK
FG07233.1 	MADQHEVDLDSIIDRLLEVRGSRPGKQVQLLEAEIRYLCTKAREIFISQPILLELEAPIK
AN0410.1  	MADQ-DVDLDSIIDRLLEVRGSRPGKQVQLLESEIRYLCTKAREIFISQPILLELEAPIK
          	***: :**************************:***************************

MG10195.1 	0ICGDIHGQYYDLLRLFEYGGFPPEANYLFLGDYVDRGKQSLETICLLLAYKIKYPENFF
NCU00043.1	0ICGDIHGQYYDLLRLFEYGGFPPEANYLFLGDYVDRGKQSLETICLLLAYKIKYPENFF
FG07233.1 	0ICGDIHGQYYDLLRLFEYGGFPPEANYLFLGDYVDRGKQSLETICLLLAYKIKYPENFF
AN0410.1  	0ICGDIHGQYYDLLRLFEYGGFPPEANYLFLGDYVDRGKQSLETICLLLAYKIKYPENFF
          	 ***********************************************************

MG10195.1 	ILRGNHECASINRIYGFYDECKRRYNIKLWKTFTDCFNCLPIAAIIDEKIFTMHGGLSPD
NCU00043.1	ILRGNHECASINRIYGFYDECKRRYNIKLWKTFTDCFNCLPIAAIIDEKIFTMHGGLSPD
FG07233.1 	ILRGNHECASINRIYGFYDECKRRYNIKLWKTFTDCFNCLPIAAIIDEKIFTMHGGLSPD
AN0410.1  	VLRGNHECASINRIYGFYDECKRRYNIKLWKTFTDCFNCLPIAAIIDEKIFTMHGGLSPD
          	:***********************************************************

MG10195.1 	LNSMEQIRRVMRPTD0IPDCGLLCDLLWSDPDKDITGWSENDRGVSFTFGPDVVSRFLQK
NCU00043.1	LNSMEQIRRVMRPTD0IPDCGLLCDLLWSDPDKDITGWSENDRGVSFTFGPDVVSRFLQK
FG07233.1 	LNSMEQIRRVMRPTD0IPDCGLLCDLLWSDPDKDITGWSENDRGVSFTFGPDVVSRFLQK
AN0410.1  	LNSMEQIRRVMRPTD0IPDCGLLCDLLWSDPDKDITGWSENDRGVSFTFGPDVVSRFLQK
          	*************** ********************************************

MG10195.1 	HDMDLICRAHQVVEDGYEFFSKRQLVTLFSAPNYCGEFDNAGAMMSVDESLL~CSFQ0IL
NCU00043.1	HDMDLICRAHQVVEDGYEFFSKRQLVTLFSAPNYCGEFDNAGAMMSVDESLL~CSFQ0IL
FG07233.1 	HDMDLICRAHQVVEDGYEFFSKRQLVTLFSAPNYCGEFDNAGAMMSVDESLL2M---~--
AN0410.1  	HDMDLICRAHQVVEDGYEFFSKRQLVTLFSAPNYCGEFDNAGAMMSVDESLL~CSFQ0IL
          	****************************************************  : .   

MG10195.1 	KPAEKKQKYVQ-RLGGRSSNASSRITSSKP
NCU00043.1	KPAEKK----------QKYVPSLR------
FG07233.1 	--AAKT----------RQSIPSPR------
AN0410.1  	KPAEKKQKYVYGAMSSGRPITPPRKQKK--
          	..* *...   .  ..    .. *  ..
```
